# Carnosol Induces p38-Mediated ER Stress Response and Autophagy in Human Breast Cancer Cells

**DOI:** 10.3389/fonc.2022.911615

**Published:** 2022-05-31

**Authors:** Halima Alsamri, Aysha Alneyadi, Khalid Muhammad, Mohammed Akli Ayoub, Ali Eid, Rabah Iratni

**Affiliations:** ^1^ Department of Biology, College of Science, United Arab Emirates University, Al Ain, United Arab Emirates; ^2^ Department of Basic Medical Sciences, College of Medicine, Qatar University Health, Qatar University, Doha, Qatar

**Keywords:** p38MAPK, ER stress, proteasome, breast cancer, ROS, unfolded protein response, autophagy

## Abstract

We recently reported that carnosol induces ROS-dependent autophagy and apoptosis in breast cancer cells. We also reported that carnosol inhibits breast cancer cell migration, invasion, and *in ovo* tumor growth, as well as targets STAT3, PCAF, and p300 to proteasome degradation. Here, we investigated the molecular mechanisms underlying its anti-malignant activity in breast cancer. We report that carnosol induces a ROS-dependent type I and type II programmed cell death (PCD-I or PCD-II, respectively), which occurred independently of each other. Indeed, chemical inhibition of autophagy had no effect on the induction of apoptosis, evident by the absence of cleaved PARP. Electron microscopy revealed that carnosol-treated cells exhibited enlarged endoplasmic reticulum, characteristic of ER stress. Markers of the three unfolded protein response pathways (PERK, IRE-1 α, and ATF6), namely ATF4, CHOP, phospho-IRE-1α, XBP1S, and cleaved ATF6 were upregulated in a ROS-dependent manner. In addition, carnosol induced a ROS-dependent activation of p38MAPK, increased the overall level of protein polyubiquitination, and targeted mTOR protein to proteasome degradation. Interestingly, inhibition of p38MAPK, by SB202190 and 203580, reduced cell death, selectively blocked the induction of IRE-1α and ATF6 UPR sensors and inhibited autophagy. In addition, inhibition of p38 reduced the carnosol-induced polyubiquitination and rescued mTOR, PCAF, and STAT3 from proteasomal degradation. Importantly, activation of PERK sensors and induction of apoptosis occurred independently of p38 activation. Taken together, our results suggest that ROS-dependent induced-ER stress contributes to carnosol-induced apoptotic and autophagic cell death in breast cancer cells, and further confirm that carnosol is a promising agent for breast cancer therapy.

## Introduction

The endoplasmic reticulum (ER) is a multifunctional organelle that is mainly responsible for protein folding and trafficking, in addition to maintaining other cellular functions. Changes in the protein-folding environment evoke accumulation of unfolded or misfolded proteins in the ER lumen, which profoundly affects several cellular processes and causes ER stress (1). This stress induces a collection of adaptive signaling pathways called an unfolded protein response (UPR) which involves the restoration of an efficient protein-folding environment and proper re-folding of misfolded protein (2). However, when ER stress is too severe to be rescued, UPR triggers autophagy and/or apoptosis (3, 4).

UPR is a highly regulated cascade system that involves three key sensors located on the ER membrane: protein kinase R-like ER kinase (PERK), transcription factor 6 (ATF6), and inositol requiring enzyme 1α (IRE1α) (2). Upon ER stress, PERK phosphorylates the alpha subunit of the eukaryotic translation initiation factor-2 (eIF2α). This, in turn, rapidly attenuates protein translation and decreases the overload of proteins in the ER lumen. This action also promotes the expression of the UPR transcription factor ATF4, which triggers apoptosis through activating the pro-apoptotic CCAAT/enhancer-binding protein-homologous protein (CHOP) leading to cell arrest or death (5, 6). CHOP can be induced by PERK/ATF4 and ATF6 (7–9), another UPR sensor. Under ER stress, ATF6 is transported to the Golgi apparatus and cleaved into fragments that can function as transcription factors. Activated ATF6 is known to regulate the expression of genes involved in the degradation of misfolded proteins (3). The third UPR sensor is IRE1, a kinase and an endoribonuclease (RNase), which upon activation, catalyzes the splicing of X-box binding protein 1(XBP1) mRNA. XBP1s is a transcription activator of essential genes known to regulate protein folding, trafficking, phospho-lipid biosynthesis, and ER membrane expansion (10, 11).

Accumulated evidence suggests that UPR signaling cascades are activated in response to oxidative stress or reactive oxygen species (ROS) accumulation (12). Abnormal increases in ROS damage cellular lipids, proteins, and DNA, resulting in irreversible oxidative damage, which ultimately leads to cell death and may culminate in pathologies like neurogenerative or cardiovascular disease (13). On the other hand, several studies have suggested the beneficial effects of ROS generation on chemotherapy-induced cell death in cancer cells (14–16). For example, bortezomib (17), apatinib (18), doxorubicin, inostamycin, vinblastin, xanthine oxidase–conjugated polymer, and camptothecin are chemotherapeutic agents that were shown to induce apoptosis by increasing ROS production (19, 20). Since some tumor cells are more sensitive to ROS than normal cells (14), the generation of ROS could be an effective approach for selectively killing cancer cells without causing significant toxicity (21, 22). Finding a new drug that stimulates ROS production and ultimately leads to UPR activation would be an attractive approach in the hunt for new effective therapeutic approaches in the fight against cancer.

A large variety of polyphenolic compounds are found in teas, vegetables, and fruits. These compounds possess different pharmacologic properties, including anticancer effects (23). Most dietary and synthetic polyphenols are known to be safe even at relatively high concentrations. Thus, polyphenols are considered promising candidates for use in developing novel anticancer drugs (24, 25). Some of these compounds exhibit their anticancer activity through ROS production and ER stress (26, 27). Moreover, preclinical studies reveal that bioactive dietary polyphenols exert anticancer effects by inducing ROS-mediated cytotoxicity in cancer cells (28).

Carnosol is a phenolic compound isolated from culinary herbs including sage, oregano, and rosemary. Currently, the interest in carnosol is on the rise due to its health-promoting properties, especially its anticancer activities in the colon (29), breast (30, 31), gastric (32) and prostate (33) cancers. Indeed, we recently showed that carnosol inhibits migration, metastasis, and tumor growth of breast cancer *via* a ROS-dependent proteasome degradation of STAT3 (31). We also reported that carnosol induces ROS-mediated beclin1-independent autophagy and apoptosis in triple-negative breast cancer (30). More recently, we showed that carnosol selectively inhibits the p300 histone acetyl transferase in breast cancer cells (34). Here, we show that carnosol triggers a ROS-dependent ER-stress response through activation of the three ER stress sensor pathways in breast cancer cells. In addition, we show that carnosol induces p38-dependent autophagy and activates the ubiquitin proteasome pathway.

## Materials and Methods

### Cell Culture, Chemicals, and Antibodies

Human breast cancer cells MDA-MB-231(cat. 300275) were purchased from Cell Line Service (CLS)-GmbH, Germany. Cells were cultured in high glucose Gibco Dulbecco’s Modified Eagle Medium (DMEM) (cat. 03640, Gibco, Life Technologies, Rockville, UK) supplemented with 10% fetal bovine serum (cat. 02187, Gibco, Life Technologies, Rockville, UK), 100 U./ml penicillin/streptomycin (cat. 01574, Gibco, Life Technologies, Rockville, UK). All Cells were maintained at 37°C under a humidified atmosphere containing 5% CO2. Carnosol (cat. C9617), and N-acetylcysteine (cat. A9165) were obtained from Sigma Aldrich (Saint-Quentin Fallavier, France). Pan-caspase inhibitor (cat. 627601) and 3-methyladenine (cat.189490) were obtained from Millipore (Hayward, CA, USA). SB 203580 (cat. Ab120162), SB 202190 (cat. Ab1206388158), and chloroquine (cat. Ab142116) were obtained from Abcam (Cambridge, UK). Bortezomib (cat. 2204) was obtained from cell signaling technologies (Beverly, MA, USA). Antibodies to phospho-mTOR (cat. 2972), total mTOR (cat. 2972), total IRE-1 (cat. 3294), XBP-1s (cat. 12782), phospho-eIF2α (cat.9721), eIF2α (cat.9722), ATF4 (cat. 11815), CHOP (cat. 2895), PDI (cat. 3501), Ero-1α (cat. 3264), phospho-p38 (cat. 4511), total p38 (cat.8690), LC3 (cat. 1274), STAT3 (cat. 9139) and ubiquitin (cat.3933) were obtained from Cell Signaling (Beverly, MA, USA). Antibodies to phospho-IRE-1 (cat Ab226974) and cleaved PARP (cat. Ab4830) were obtained from Abcam (Cambridge, UK). Antibody to cleaved ATF6 (cat. A12570) was obtained from AB clonal (Woburn, MA, USA). Antibodies to PCAF (cat. Sc13124) antibody, β-actin (Sc-47778), goat anti-mouse IgG-HRP (Cat. # sc-2005), and goat anti-rabbit IgG-HRP antibody (Cat. # sc-2004) were from Santa Cruz Biotechnology Inc (USA).

### Cellular Viability

Cells were seeded in triplicate in 12-well plates at a density of 50,000 cells/well and left in culture for 24 hours before treatment with or without carnosol for 24 hours. Cellular viability was measured with the Muse Cell Analyzer (Millipore, Hayward, CA, USA) using the Muse Count and Viability Kit (Millipore, Hayward, CA, USA) which differentially stains viable and dead cells based on their permeability to two DNA binding dyes. Data were presented as proportional viability (%) by comparing the treated vs. the untreated cells, the viability of which is considered to be 100%.

### Analysis of the Mitochondrial Membrane Potential

Cells (50,000) grown in 12-well plates for 24 hours were first treated with or without carnosol for 24 h in the presence or absence of the indicated inhibitors, collected by trypsinization, washed, and incubated with the Muse Mitopotential Dye (Muse MitoPotential kit, Millipore), a cationic lipophilic dye, for 20 min in a 37°C CO2 incubator. Cells were then incubated with 7-AAD, a dead cell marker, for an additional 5 min at room temperature. The mitochondrial membrane potential changes were determined using the Muse Cell Analyzer (Millipore).

### Transmission Electron Microscopy

Transmission electron microscopy (TEM) was carried out as previously described (REF carnosol paper PLOS one). Briefly, Control and carnosol-treated cells were fixed in fixation buffer (2% paraformaldehyde, 2.5% glutaraldehyde in 0.1 M sodium cacodylate, pH 7.2) overnight at 4°C, before being post-fixed with 1% OsO4 for 1 h. Cells were then dehydrated in a graded ethanol series and embedded in Agar 100 epoxy resin. Ultrathin sections were mounted on Cu grids and stained first with uranyl acetate followed by lead citrate. Sections were observed and photographed under a Philips CM10 Transmission Electron Microscope.

### Whole Cell Extract and Western Blotting Analysis

Breast cancer cells (1.8 ×10^6^) were seeded in 10 cm tissue culture dishes and left in culture for 24 hours. Cell s were then treated with or without carnosol in the presence or absence of N-acetylcysteine, SB 203580, or SB202190 for another 24 hours. Cells were washed twice with ice-cold PBS, scraped, and collected by centrifugation at 2000 rpm for 5 min. The pelleted cells were then lysed in RIPA buffer (ThermoFisher Scientific) containing protease inhibitor cocktail (Roche) and phosphatase inhibitor (Roche) followed by incubation for 30 min on ice. Cell lysates were centrifuged at 14,000 rpm for 20 min at 4°C, and the supernatants were collected. Total protein concentration was quantified by a BCA protein assay kit (ThermoFisher Scientific) and the lysates were adjusted with lysis buffer. Aliquots of 20 μg of total cell lysate were loaded and resolved onto 6–15% SDS-PAGE. After electrophoresis, proteins were transferred from the gels to PVDF membranes (Thermo Scientific) and blocked for 1 h at room temperature with 5% non-fat dry milk in TBST (TBS and 0.05% Tween 20). The membranes were immunoblotted with specific primary antibodies in blocking buffer overnight at 4°C and then with horseradish peroxidase-conjugated secondary antibodies against rabbit or mouse IgG. Immunoreactive protein bands were detected, depending on the targeted protein, by ECL chemiluminescent substrate (ThermoFisher Scientific) or SuperSignal™ West Femto Maximum Sensitivity Substrate (ThermoFisher Scientific) and, chemiluminescence was detected using the LiCOR C-DiGit blot scanner (LI-COR Biosciences). Quantification was carried using the ImageJ software.

### Statistical Analysis

Data were presented as means ± S.E.M. Differences between groups were analyzed using a Student’s t-test for paired or unpaired values. Graphpad prism (v.6.0) software was used for statistical analysis. P- values of <0.05 were considered to be statistically significant. Unless otherwise stated, experiments were repeated at least 3 times.

## Results

### Autophagic and Apoptotic Cell Death Occurs Independently From Each Other in Carnosol-Treated Breast Cancer Cells

**Figure 1:**
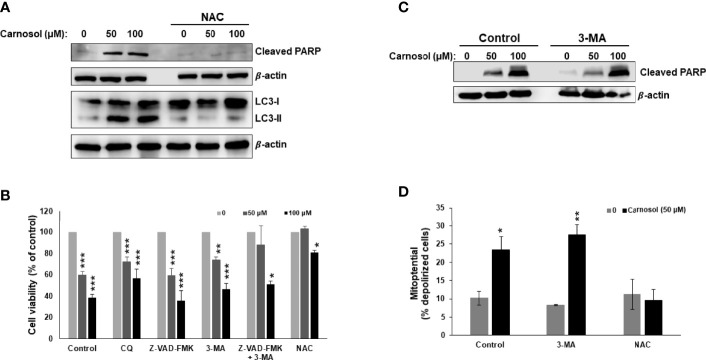
Carnosol induces breast cancer cell death through activation of PCD-I and PCD-II pathways. **(A)** ROS-dependent LC3II accumulation and PARP cleavage in carnosol-treated MDA-MB-231 cells. Cells were pre-treated with the ROS scavenger NAC (10mM) before adding carnosol at the indicated concentrations for 24 hours. Whole cell lysates were analyzed by Western blot for LC3II and cleaved PARP. **(B)** Inhibition of autophagy but not apoptosis reduces carnosol-induced cell death. MDA-MB-231cells were pretreated with the inhibitors of autophagy CQ (50 μM) and 3-MA (10mM), or the pan-caspase inhibitor (Z-VAD-FMK) (50 μM) alone or in combination then treated with carnosol for 24 hours. Cell viability was determined as described in Material and Methods. **(C)** Western blot of cleaved PARP in cells pretreated with and without autophagy inhibitor. **(D)** Inhibition of autophagy failed to restore the carnosol-induced loss of mitochondrial membrane potential in MDA-MB-231 cells. Mitochondrial membrane potential (MMP) of cells pre-treated with 3-MA or NAC, prior to the addition of carnosol for 24 hours, was assessed with the Muse Cell Analyzer using the Muse MitoPotential kit as described in Materials and Methods. Data represent the mean ± SEM of at least 3 independent experiments. Student’s t-test was performed to determine the significance (*p<0.05, **p<0.005, and ***p<0.001).

We have previously reported that carnosol induced ROS-dependent autophagy and apoptosis in MDA-MB-231 breast cancer cells (30). While autophagy was triggered as early as 6 h post-treatment, apoptosis was detected later. Because both events are known to induce cell death through PCD-I and PCD-II, respectively, we decided to extend our previous work and investigate the contribution of these two events (apoptosis and autophagy) to the cytotoxic activity of carnosol. We first confirmed, by Western blotting, the ROS-dependent induction of apoptosis and autophagy in carnosol-treated cells (**Figure 1A**). Next, we examined the effect of chloroquine (CQ) and 3-methyadenine (3-MA), two autophagy inhibitors, and Z-VAD-FMK, a pan-caspase inhibitor, on cell viability. As shown in **Figure 1B**, cell viability was modestly but significantly improved when autophagy was inhibited compared to control cells treated with carnosol alone. On the other hand, inhibition of apoptosis by Z-VAD-FMK had almost no effect on cell death when compared to cells treated with carnosol alone (**Figure 1B**). Interestingly, a combination of both inhibitors (3-MA and Z-VAD-FMK), restored cell viability, at 50 µM carnosol, to a level comparable to control cells. A high concentration of carnosol (100 µM) led to only a modest recovery of cell viability (**Figure 1B**). As expected, and as previously reported, inhibition of ROS generation by N-acetylcysteine (NAC) completely abolished the cytotoxic effect of carnosol even at high concentration (**Figure 1B**). The fact that cell death still occurred even when autophagy or apoptosis was inhibited, suggests that these two mechanisms of cell death are independent of each other. This is further confirmed by the detection of cleaved PARP (a marker of apoptosis) in cells where autophagy was inhibited by 3-MA (**Figure 1C**). Similarly, inhibition of autophagy by 3-MA did not affect the carnosol-induced loss of mitochondria membrane potential (**Figure 1D**). Taken together, our data suggest that in carnosol-treated cells, autophagy and apoptosis are independent events and both lead to cell death and that one mechanism may indeed compensate for the other.

### Carnosol Induces Proteasome-Dependent Degradation of mTOR in Breast Cancer Cells

**Figure 2:**
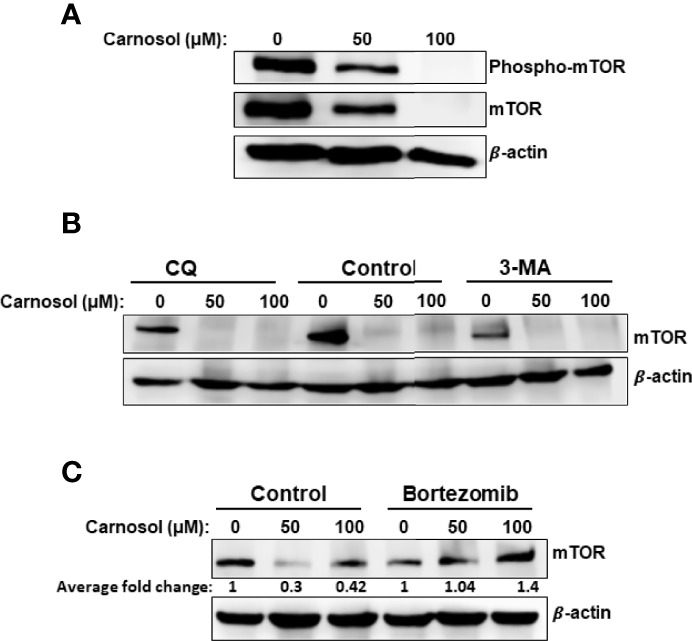
Targeting of mTOR protein to ROS-dependent proteasome degradation. **(A)** Inhibition of mTOR signaling by carnosol in MDA-MB-231 cells. Cells were treated for 24 hours with carnosol (50 and 100 μM) and whole protein lysates were analyzed by Western blot for phosphorylated and total mTOR protein. **(B)** Western blot analysis of mTOR protein level in MDA-MB-231 cells pre-treated with the autophagy inhibitors 3-MA and CQ. Cells were pretreated with or without 3-MA (50 mM) and CQ (50 μM) for 1 h before adding carnosol for 24 hours. **(C)** Carnosol targets mTOR to proteasome degradation. MDA-MB-231 cells were pre-treated for 1 h with or without Bortezomib (25 nM) before treatment with carnosol at the indicated concentrations for 24. Whole protein lysates were resolved on SDS-PAGE and analyzed for mTOR protein.

We next examined the effect of carnosol on the mTOR pathway, a major negative regulator of autophagy. This is of particular interest because mTOR signaling is known to be dysregulated in many human cancers, including breast cancer. We first determined the level of phospho-mTOR(ser2448). As shown in **Figure 2A**, carnosol reduced, in a concentration-dependent manner, the level of phosphorylated mTOR, indicating that mTOR pathway is inhibited in treated cells. Strikingly, we also found that carnosol induced a significant decrease in the level of total mTOR (**Figure 2A**). Since carnosol induced autophagy, we first sought to determine whether STAT3 was targeted to autophagolysosome degradation. Our interesting results show that blockade of autophagy by 3-MA or CQ did not restore the level of mTOR protein (**Figure 2B**), hence excluding autophagy as a mechanism of degradation of mTOR protein. Next, we tested whether mTOR was a target of proteasomal degradation. We found that bortezomib, a proteasome inhibitor, restored the mTOR protein to a level comparable with that of control cells (**Figure 2C**). This clearly indicates that carnosol targets mTOR to proteasome degradation, ultimately leading to the activation of autophagy.

### Carnosol Induces a ROS-Dependent Activation of the UPR and Increase in Protein Polyubiquitination in Breast Cancer Cells

**Figure 3:**
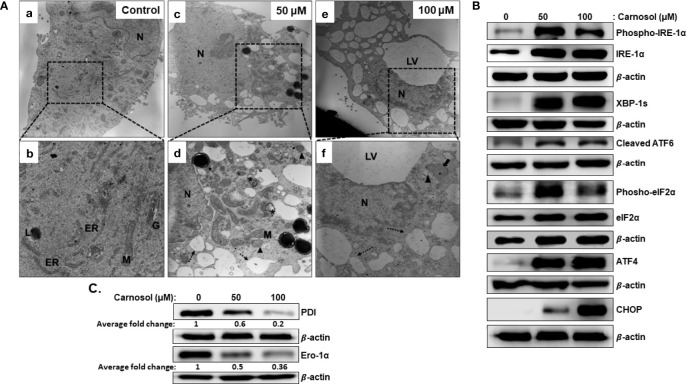
Carnosol induces ER stress in MDA-MB-231 cells. **(A)** Representative electron micrographs of untreated MDA-MB-231 cells **(a–b)** and MDA-MB-231 cells treated with 50 **(c–d)** and 100 µM **(e–f)** carnosol for 24 h. **(B)** Activation of the UPR sensors in carnosol-treated cells. Cells were treated for 24 hours with carnosol (50 and 100 μM) and whole protein lysates were analyzed by Western blot for proteins of the ER stress markers. **(C)** Carnosol disrupts the proper protein folding machinery. Immunoblot of protein folding enzymes PDI and Ero-1α. Protein extract was prepared as described in **(B)**.

We have previously shown that carnosol induced mitophagy in MDA-MB-231 cells (30). Further examination of ultrastructural changes *via* transmission electron microscopy of carnosol-treated cells revealed many dilated endoplasmic reticula (**Figure 3A**, panel d–f, dashed arrow). In addition, multilamellar ER structures are also observed (**Figure 3A**, panel d, asterisk). Similar to our previous report, damaged mitochondria can also be seen (**Figure 3A**, panel d–f, arrowhead). Several autophagolysosomes could be seen in treated cells (**Figure 2A**, panel d, thin arrow). The large number of swollen ER prompted us to hypothesize that this effect is triggered by carnosol-induced ER stress. To test our hypothesis, we assessed the activation of PERK, ATF6, and IRE-1α pathways. Our results show that carnosol induced phosphorylation of IRE-1 α and EIF2 α, and increased levels of XBP-1s, cleaved ATF6, ATF4, and CHOP (**Figure 3B**). These results suggest that the three UPR pathways were activated by carnosol. We next examined the protein level of protein disulfide isomerase (PDI) and endoplasmic reticulum oxidoreductase 1 (ERO-1α), two enzymes that promote proper protein folding in the ER and are upregulated in several cancers including breast cancer. We found that carnosol caused a dramatic decrease in the level of PDI and ERO-1α enzymes (**Figure 3C**), hence further suggesting an impaired protein folding of the ER.

**Figure 4:**
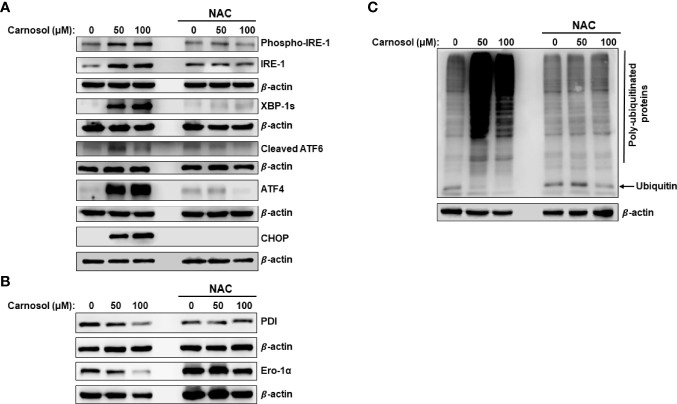
ROS-dependent induction of ER stress and increase in protein ubiquitination. **(A, B)** ROS-dependent activation of UPR sensors in carnosol-treated MDA-MB-231 cells. Cells were pre-treated with the ROS scavenger NAC (10mM) before adding carnosol at the indicated concentrations for 24 hours. Whole cell lysates were analyzed by Western blot for ER stress markers **(A)** and protein folding enzymes **(B)**. **(C)** Carnosol increases the cellular level of polyubiquitinated proteins in MDA-MB-231 cells. Cells were treated with or without carnosol (50 and 100 µM) for 24 h, then whole-cell extracts were subjected to Western blot analysis for ubiquitinated proteins.

We have shown that carnosol induces a ROS-dependent cytotoxic effect on MDA-MB-231 cells (**Figure 1A**). Pre-treatment of MDA-MB-231 cells with NAC (**Figure 1A**) a ROS scavenger, abolished the carnosol-mediated cytotoxic effect, thus demonstrating that carnosol exerts its anticancer activity through a ROS-dependent manner. This prompted us to examine whether carnosol-induced ER stress is ROS-dependent. As shown in **Figure 4A**, NAC completely abolished the activation of PERK, IRE1α, and ATF6, strongly suggesting that induction of ER stress is solely dependent upon the carnosol-mediated generation of ROS. Similarly, carnosol failed to decrease the level of PDI and ERO-1α in the presence of NAC (**Figure 4B**), hence arguing in favor of the normal protein folding function of ER when ROS accumulation is inhibited.

It is documented that impaired folding function of ER is associated with increased protein polyubiquitination. Other studies also showed that inhibition of mTOR activity increases the overall protein ubiquitination and degradation by the ubiquitin proteasome system (UPS) (35). This prompted us to measure the overall level of protein ubiquitination in carnosol-treated cells. As shown in **Figure 4C**, carnosol induced a dramatic increase in the overall level of polyubiquitinated proteins, an effect that was completely abolished by NAC.

### Carnosol Induces p38-Dependent Autophagy in Breast Cancer Cell

**Figure 5:**
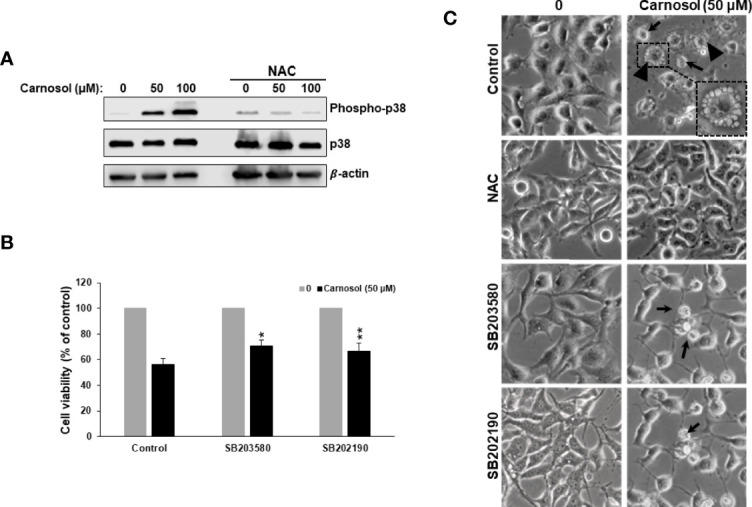
ROS-dependent activation of p38MAPK by carnosol. **(A)** Concentration-dependent increase of phospho-p38 protein in carnosol-treated MDA-MB-231 cells. Cells were pre-treated with the ROS scavenger NAC (10mM) before adding carnosol at the indicated concentrations for 24 hours. Whole cell lysates were analyzed by Western blot for phospho-p38, total p38, and β-actin (loading control). **(B)** Chemical inhibition of p38 activation reduced carnosol-induced cell death. MDA-MB-231 cells were pre-treated for 1 h with or p38 inhibitors, SB203580 (50 µM), or SB202190 (50 µM) before treatment with carnosol (50 µM) for 24 hours. Cell viability was determined as described in Material and Methods. Data represent the mean ± SEM of 3 independent experiments. The student’s t-test was performed to determine the significance (*p<0.05 and **p<0.005). **(C)** Morphological changes in carnosol-treated MDA-MB-231 cells. Cells were observed under EVOS XL Core Cell Imaging System (Life Technologies) at 400X.

p38MAPK is involved in ER stress, autophagy, and apoptosis in many types of cancer cells. This prompted us to examine p38 activation in carnosol-treated cells. As shown in **Figure 5A**, carnosol induced a concentration-dependent increase in the level of phosphorylation of p38. Next, we investigated the role of p38 activation in carnosol-mediated ER stress, autophagy, and apoptosis. Toward this, we found that SB203580 and 202190, two p38 inhibitors, significantly promoted cell viability (70% and 67%, respectively) (**Figure 5B**) when compared to control cells treated with carnosol only (56%), thus suggesting that p38MAPK contributes to carnosol-induced cell death. Since both autophagy and apoptosis contribute to carnosol-induced cell death (**Figure 1**), we wanted to examine which mechanism is affected by p38. We first looked at the morphology of MDA-MB-231 cells treated with carnosol alone or in a combination of carnosol and p38 inhibitors. As shown in **Figure 5C**, light microscopy observation of cells treated with carnosol alone revealed cytoplasmic vacuolation, indicative of autophagy, in a subpopulation of treated cells (arrowheads). Also, smaller and rounded cells, characteristic of apoptotic cells were observed (thin arrows). Interestingly, no autophagic vacuoles were seen when in cells treated with both carnosol and p38 inhibitors (**Figure 5C**). However, cells with morphological characteristics of apoptosis were observed (thin arrows) in the presence of p38 inhibitors. As expected, inhibition of ROS generation by NAC completely abolished the morphological changes associated with carnosol treatment (**Figure 5C**). Altogether, these data supported our hypothesis that p38 activation might be involved in the induction of autophagy but not apoptosis.

**Figure 6:**
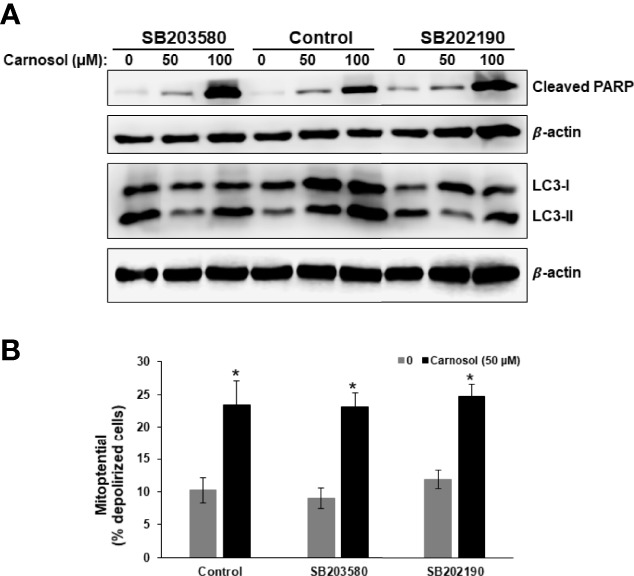
p38-dependent induction of autophagy in carnosol-treated MDA-Mb-231 cells. **(A)** Inhibition of p38 blocks carnosol-induced autophagy in MDA-MB-231 cells. Cells were pre-treated with the p38 inhibitors (SB203580 or SB202190) before adding carnosol (50 and 100 µM) for 24 hours. Whole cell lysates were analyzed by Western blot for the marker of autophagy (LC3II) and apoptosis (cleaved PARP). **(B)** Inhibition of p38 activation failed to restore the carnosol-induced loss of mitochondrial membrane potential in MDA-MB-231 cells. Mitochondrial membrane potential (MMP) of cells pre-treated with p38 inhibitor, prior to the addition of carnosol for 24 hours, was assessed with the Muse Cell Analyzer using the Muse MitoPotential kit as described in Materials and Methods. Data represent the mean ± SEM of at least 3 independent experiments. Student’s t-test was performed to determine the significance (*p<0.05).

To confirm this hypothesis, we examined the level of LC3II and cleaved PARP, markers of autophagy and apoptosis, respectively, in cells treated with carnosol in the absence or presence of p38 inhibitors. As shown in **Figure 6A**, inhibition of p38 did not have any effect on the level of cleaved PARP. Similarly, inhibition of p38 did not affect the carnosol-induced loss of mitochondria membrane potential (**Figure 6B**). These results reveal that carnosol-induced apoptosis is p38-independent. On contrary, inhibition of p38 evoked a dramatic decrease in the level of the autophagy marker, LC3II (**Figure 6A**). This finding along with the morphological observation (absence of cytoplasmic autophagic vacuolation) (**Figure 5C**) demonstrates that induction of autophagy is p38-dependent.

### Carnosol Activates the IRE1α and ATF6 ER Stress Pathways *via* a p38-Dependent Mechanism

**Figure 7:**
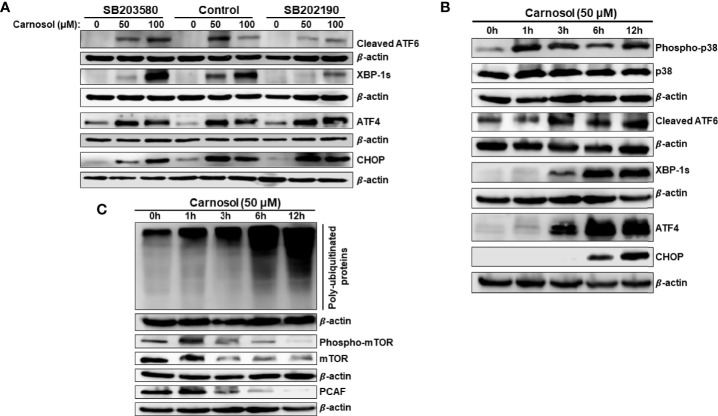
p38MAPK activation of ATF6 and IRE-1α UPR sensors. **(A)** Chemical inhibition of p38 blocked the activation of ATF6 and IRE-1α UPR sensors but did not affect the activation of the PERK sensor. Cells were pre-treated with the p38 inhibitors (SB203580 or SB202190) before adding carnosol (50  µM) for 24 hours. Whole cell lysates were analyzed by Western blot for markers of ER stress (cleaved ATF6, XBP-1s, ATF4, and CHOP). **(B)** Time-course analysis, by Western blotting, of phospho-p38, total p38, cleaved ATF6, XBP-1s, ATF4, and CHOP. Cells were treated with carnosol (50 μM) and proteins were extracted at the indicated time points (0, 1, 3, 6, 12  hours) as described in Materials and Methods. **(C)** Time-course analysis, by Western blotting, of phospho-mTOR, total mTOR, and PCAF.

Next, we investigated the role of p38 activation in carnosol-induced ER stress response. We assessed the level of cleaved ATF6, XBP-1s, ATF4, and CHOP in cells treated with carnosol in the absence or presence of SB203580 or SB202190. **Figure 7** reveals that blocking p38 inhibited the activation of ATF6 and the expression of XBP-1s. Interestingly, both inhibitors did not modulate the activity of PERK sensors, ATF4 and CHOP. This indicates that p38 selectively activates the ATF6/IRE1α ER stress pathway.

We have previously reported that ROS generation occurs as early as 1 hour while autophagy and apoptosis occurred at 6 hours and 24 hours post-carnosol treatment, respectively (30). Here, we show that p38 activation also occurred as early as 1 hour post-carnosol treatment (**Figure 7B**). On the other hand, activation of ER stress sensors was observed later starting from 3 hours post-treatment (**Figure 7B**). It is worth mentioning that CHOP expression was delayed, detected at 6 h, compared to its upstream inducer ATF4, detected at 3 hours showed (**Figure 7B**). This result further cements the argument that p38 activation precedes UPR response in carnosol-treated cells.

Next, we determined the time at which protein polyubiquitination and mTOR degradation occurred. An increase in the overall protein polyubiquitination was as well as mTOR degradation was detectable as early as 3 hours post-carnosol treatment (**Figure 7C**). Because we had previously shown that carnosol also induced a proteasome degradation of STAT3 (31)as well as p300 (34)and PCAF (34) histone acetyltransferases, we decided to determine the time at which this degradation occurs. As shown in **Figure 7C**, similar to mTOR’s, PCAF degradation was detected as early as 3 hours post-treatment.

Based on these data along with our previous findings, we can stipulate that the sequence of events induced in breast cancer by carnosol is as follows: ROS generation, p38 activation, ER stress concomitant with polyubiquitination and protein degradation, then autophagy followed by apoptosis.

### p38 Promotes Protein Degradation in Carnosol-Treated Breast Cancer Cells

**Figure 8:**
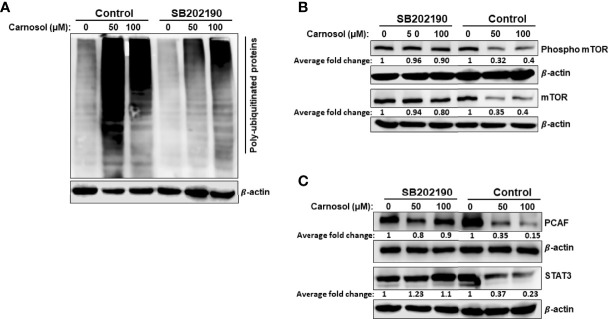
Inhibition of p38MAPK reduces carnosol-mediated protein polyubiquitination and proteasome degradation of mTOR, STAT3, and PCAF. **(A)** Inhibition of p38 reduces the level of polyubiquitinated proteins in MDA-MB-231 cells. Cells were pre-treated for 1 h with or without SB202190 (50 μM) before treatment with carnosol (50 µM). Whole cell lysates were subjected to Western blotting analysis for ubiquitinated proteins. **(B, C)** SB202190 restored mTOR PCAF and STAT3 proteins to a level comparable to control cells. Cells were treated as described in **(A)** and whole protein lysates were subjected to Western blotting analysis for mTOR and phospho-mTOR **(B)** and PCAF and STAT3 **(C)**.

We have shown that activation of p38 (**Figure 7B**) precedes protein ubiquitination and protein degradation (**Figure 7C**). Therefore, we next wished to investigate whether these last two events were tied to p38 activation. To this end, we first analyzed the protein ubiquitination level in cells treated with carnosol (50 μM) in the presence or absence of SB202190. Our results show that inhibition of p38 activation dramatically reduced protein ubiquitination (**Figure 8A**), and almost completely rescued mTOR from degradation (**Figure 8B**). Interestingly, the level of phosphorylated mTOR was also restored to a level comparable to that in control untreated cells (**Figure 8B**). These findings are in agreement with the inhibition of autophagy by the p38 inhibitors. Altogether, these results strongly suggest that p38 activation, in response to carnosol-induced ROS generation, induces autophagy by targeting mTOR for proteasomal degradation.

Having shown that inhibition of p38 prevented the proteasomal degradation of mTOR protein, we decided to investigate whether STAT3 and PCAF proteasomal degradation was mediated through a p38-dependent mechanism. Indeed, treatment with SB202190 efficiently prevented STAT3 and PCAF degradation by the proteasome (**Figure 8C**), hence strongly suggesting that p38 activation contributes to the induction of the ubiquitin proteasome system leading to degradation of proteins like mTOR, p300, PCAF, and STAT3.

## Discussion

We have previously reported that carnosol induced a ROS-dependent beclin-1 independent autophagy and apoptosis in MDA-MB-231 breast cancer cells (30). However, the relationship between these two events in carnosol-mediated cytotoxicity was not investigated. Here, we extended the work and examined whether these two mechanisms of cell death were linked. Here, we showed that autophagy and apoptosis are triggered *via* ROS-mediated ER stress and that both mechanisms contribute to carnosol-induced cell death independently of each other. Interestingly, we found that autophagy is induced *via* the p38-mediated induction of the IRE-1α and ATF6 pathways, while apoptosis seems to be activated through the PERK-ATF4-CHOP pathway. We also found that activation of p38MAPK leads to an increase in protein polyubiquitination and activation of the ubiquitin proteasome system causing the degradation of several proteins including mTOR, p300, PCAF, and STAT3. Inhibition of p38MAPK reduced carnosol-induced cytotoxicity, inhibited the induction of IRE-1α and ATF6 and autophagy and blocked protein degradation. Further analysis revealed that p38MAPK activation preceded the induction of autophagy and UPS activation in carnosol-treated cells.

PCD-I (Apoptosis) and PCD-II (autophagy) are two different mechanisms of cell death and cross-talk between them exists. It is also very common that autophagy and apoptosis usually occur within the same cell, where autophagy precedes apoptosis. The intricate interplay between these two mechanisms of cell death is a major challenge for cancer treatment. The relationship between autophagy and apoptosis in cancer cells is quite complex and can be divided into synergistic/collaborative, promoting or antagonistic effects depending on the cell type and, nature and duration of the stress (35). Carnosol induced both beclin-1 independent autophagic and apoptotic cell death of breast cancer cells. Inhibition of autophagy had only a very limited effect on carnosol-induced cell death. Also, apoptosis still occurred even when autophagy was inhibited. Moreover, blocking autophagy failed to reduce ROS generation or restore the mitochondrial membrane potential. On the other hand, inhibiting apoptosis did not prevent carnosol-induced cell death. These findings suggest that the two mechanisms are activated concomitantly through different mechanisms and might act collaboratively to promote cell death of breast cancer cells, particularly in response to carnosol-induced cellular damage. Because apoptosis is triggered only 24 hours post-carnosol treatment, while autophagy was detectable as early as 6 hours post-treatment, we believe that cell death through activation of apoptosis comes as a secondary response due to increased intracellular stresses and therefore increased cellular damage due to longer exposure of the cells to carnosol. Like carnosol, an increasing number of anticancer drugs have been reported to induce autophagic and apoptotic cells death of different cancer cell types through synergistic/collaborative effect. For example, dovitinib, a novel multi-target receptor tyrosine kinase inhibitor enrolled in several clinical trials in different cancers, was shown to induce autophagic cell death and apoptosis in several breast cancer cell lines including MDA-MB-231 (36). Dovitinib was shown to mediate its cytotoxic effect through inhibition of STAT3. Interestingly, we have previously shown that carnosol inhibited the STAT3 pathway through targeting STAT3 protein to proteasome degradation. Hence, we cannot exclude that the downregulation of the STAT3 pathway could also account, at least partly, for the induction of PCD-1 and PCD-II in carnosol-treated breast cancer cells. Tetraarsenic hexoxide (As_4_O_6_) was also shown to induce p38-mediated beclin1-independent autophagic and apoptotic cell death in SW620 colon cancer cells (37). For example, berberine was shown to induce both apoptotic and autophagic death of HepG2 cancer cells (38).

The ER plays many crucial cellular functions such as protein folding and secretion, lipid biosynthesis, and calcium homeostasis. In addition, the ER is also involved in oxygen and nutrient sensing as cells adapt to their microenvironment (39). Disturbance of ER function by various stressors such as oxidative stress, DNA damage, nutrient deficiency, or calcium depletion can lead to ER stress with subsequent accumulation of unfolded or misfolded proteins which ultimately triggers UPR to restore metabolic homeostasis (27, 40). However, under persistent ER stress, components of the UPR pathways, namely ATF4, XBP-1s, and CHOP can also lead to cell death through activation of PCD-I, PCD-II, or both (27).

The increasing number of natural compounds are known to trigger a ROS-dependent ER stress-mediated cell death in several types of cancer including breast cancer cells (26). One such compound, saxifragifolin D (SD) isolated from* Androsace umbellate*, was shown to inhibit breast cancer by inducing ROS-dependent ER stress leading to autophagy and apoptotic cell death (41). SD-induced ER stress was shown to be associated with the upregulation of IRE-1α expression, and an increase in the level of XBP-1s, BiP and CHOP proteins (41). Another natural compound, γ-tocotrienol and member of the vitamin E, was also shown to promote breast cell death through induction of ER stress, autophagy, and apoptosis (42, 43). Indeed, γ-tocotrienol-induced cell death of MDA-MB-231 and MCF-7 breast cancer cells was partially reduced by inhibition of either autophagy or apoptosis (43). γ-tocotrienol also induced markers of ER stress through modulation of the UPR markers BiP, phospho-PERK, phospho-EIF2α, phsopho-IRE1α, ATF3, ATF4, and CHOP (42, 43). Interestingly, γ-tocotrienol-induced apoptosis might involve the ATF4-ATF3-CHOP pathway (42). Here we showed that carnosol induces a ROS-dependent ER stress through activation of the three main UPR pathways, namely PERK, ATF6, and pIRE-1α. We also showed that both autophagy and apoptosis contribute through an independent mechanism to carnosol-induced cell death. Interestingly, we reported here that only the PERK-ATF4-CHOP pathway of the UPR seems to be responsible for the induction of apoptosis in carnosol-treated cells. Indeed, inhibiting p38, which blocked the activation of ATF6 and pIRE-1α pathways and autophagy, had no effect on the PERK pathway nor the activation of the apoptotic program demonstrated by PARP cleavage. It is well-documented that prolonged activation of the UPR might initiate apoptotic cell death through the upregulation of CHOP, which indeed plays a role in ER stress-mediated apoptosis (44). Although CHOP could be induced by the three UPR pathways, we believe that in the presence of carnosol, only the PERK-ATF4 contributes to CHOP upregulation. This is supported by the observation that the inhibition of ATF6 and IRE1-α pathways, by SB202190 or SB203580, did not affect the upregulation of carnosol-induced CHOP expression. This is strongly indicative that activation of apoptosis depended mainly on the PERK-ATF4-CHOP pathway. It is noteworthy to mention that one mechanism through which CHOP induces apoptosis is *via* downregulation of the expression of the anti-apoptotic proteins BcL2, BcL-xL, and Mc-1 and upregulation of pro-apoptotic proteins such as Bax, Bim and BAK (44). Interestingly, we have previously reported that carnosol downregulated BcL-2 while upregulated Bax protein in MDA-MB-231 breast cancer cells (30).

p38 links extracellular signals to the intracellular machinery that regulates a plethora of cellular processes that including regulation of the cell cycle, induction of cell death, differentiation, and senescence depending on the kinetics of activation and downstream signaling pathways activated (45–47). Recent studies showed that p38MAPK plays a dual role in the unfolded protein response pathway (48, 49). p38MAPK can be activated as a consequence of UPR activation through IRE-1α and PERK oligomerization (49, 50). Furthermore, sustained activation of p38 MAPK induces ER stress and activates the UPR response pathway by regulating the expression of UPR components such as BiP (49, 51), CHOP (52), ATF6 (53) and XBP-1s (54). Studies showed that in the presence of ER stress, p38MAPK can promote cell death by autophagy and/or apoptosis of various types of cancer depending on the type and duration of the stimulus. Here we showed that p38MAPK was not a component of a signaling pathway that led to the activation of apoptosis. Indeed, apoptosis occurred in carnosol-treated cells even when p38 activation was inhibited, hence excluding p38 in the activation of PCD-I. However, our data strongly suggest that p38MAPK activation, by a ROS-dependent mechanism, triggers selective activation of IRE-1α and ATF6 UPR response pathway, followed by stimulation of the ubiquitin proteasome system (UPS) and induction of autophagy. Time course analysis showed that activation of p38, is the first event that occurred in carnosol-treated cells followed by the concomitant activation of IRE-1α and ATF6 and UPS, and then by autophagy. Inhibition of p38 blocked the carnosol-induced activation of IRE-1α and ATF6 sensors without affecting the CHOP activation, decreased UPS activity and blocked autophagy, as well as attenuated cell death in breast cancer cells. These findings strongly suggest that, under certain stress conditions, p38 plays a stimulatory role in the ubiquitin proteasome system. We also propose that induction of autophagy is probably a consequence, although another mechanism may be involved, of the proteasomal degradation and hence inactivation of the negative regulator of autophagy, mTOR protein. This is supported by the notion that degradation of mTOR, which occurred as early as 3 hours post-carnosol treatment, preceded autophagy which was detectable only after 6 hours. Also, blockade of proteasome activity not only restored total mTOR protein level but also restored phosphorylated mTOR which restored its ability to inhibit autophagy. mTOR inhibition was shown to induce autophagy and stimulate protein breakdown (55). A recent study showed that mTOR inhibition by Torin 1 or rapamycin stimulates and enhances overall protein degradation by the ubiquitin proteasome system as well as by autophagy in HEK 293 cells (56). Another study showed that *Rhus coriaria* ethanolic extract induced autophagy and increased the overall level of protein ubiquitination and the degradation of several proteins including mTOR protein in HT-29 colon cancer cells (57). The mechanism through which inhibition of mTOR stimulates proteasome degradation remains poorly understood. Our current data further confirm the role of mTOR inhibition in stimulating the UPS activity but also incriminates p38 MAPK in this process.

**Figure 9:**
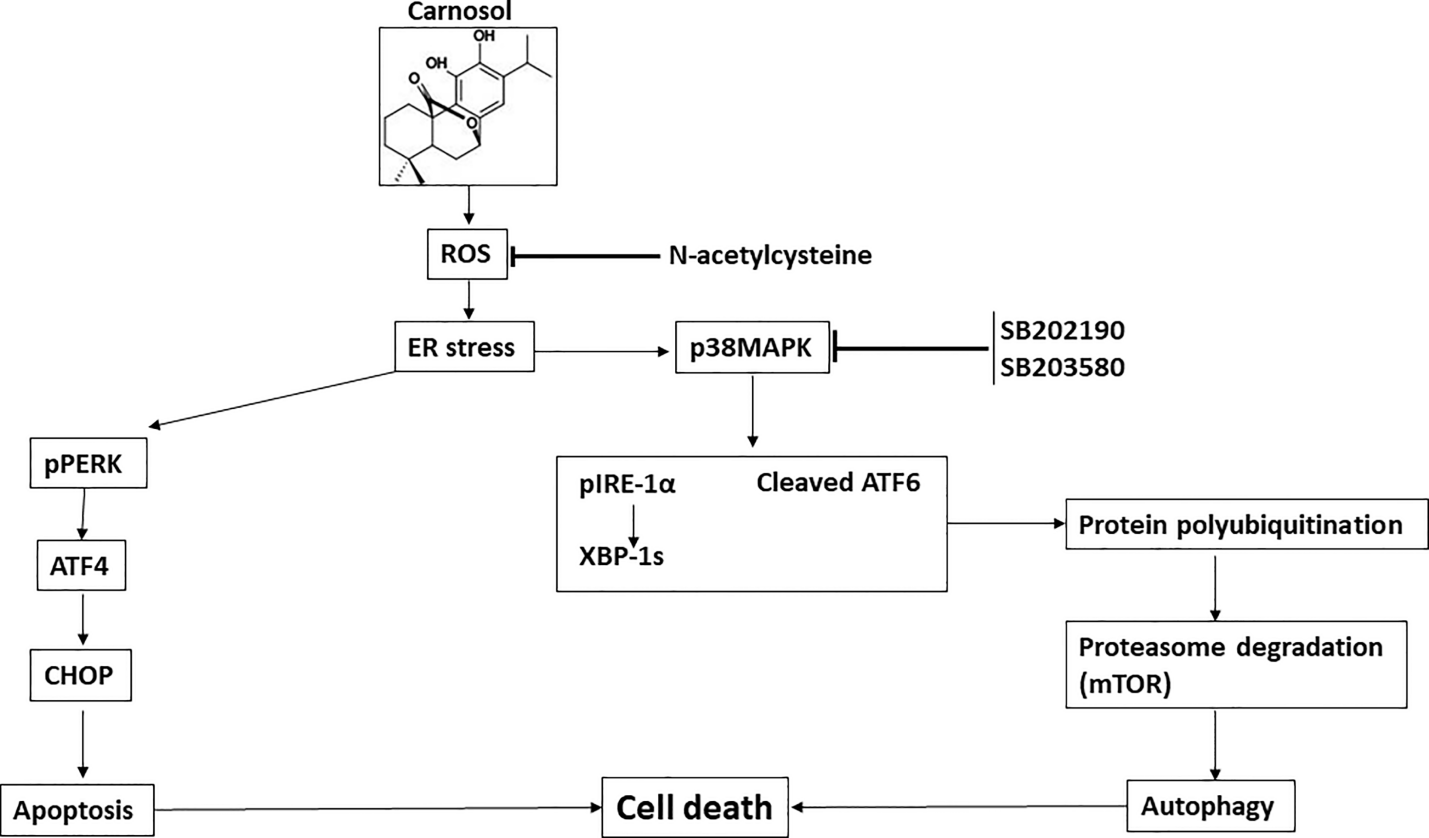
Hypothetic model for the anti-breast cancer effect of carnosol.

In summary, our current findings further enhance our understanding of the molecular mechanisms through which carnosol exerts its anticancer activity at least in the case of breast cancer. We show for the first time that carnosol induces a ROS-dependent activation of p38 MAPK which in turn activates ER stress response IRE-1a and ATF6 associated with stimulation of the proteasome activity leading to the degradation of the autophagy regulator mTOR. As consequence, autophagy is activated leading to cell death. We also show, that apoptosis is triggered through a p38MAPK-independent pathway, yet to be uncovered. Hence, our data are consistent with the model shown in **Figure 9**. These current findings along with previous reports provide further evidence that carnosol is a good candidate for treating an aggressive form of breast cancer.

## Data Availability Statement

The raw data supporting the conclusions of this article will be made available by the authors, without undue reservation.

## Author Contributions

HA, AA, KM, and MA performed all experiments. AE edited the manuscript. RI designed the project, analyzed the data, and wrote the manuscript. All authors reviewed the manuscript. All authors contributed to the article and approved the submitted version.

## Funding

This work was supported by Al Jalila Foundation (Grant 21S102-AJF2018007) and by the Zayed Center for Health Sciences (ZCHS) research grant (Grant # 31R086).

## Conflict of Interest

The authors declare that the research was conducted in the absence of any commercial or financial relationships that could be construed as a potential conflict of interest.

## Publisher’s Note

All claims expressed in this article are solely those of the authors and do not necessarily represent those of their affiliated organizations, or those of the publisher, the editors and the reviewers. Any product that may be evaluated in this article, or claim that may be made by its manufacturer, is not guaranteed or endorsed by the publisher.
